# A selective antagonist of prostaglandin E receptor subtype 4 attenuates abdominal aortic aneurysm

**DOI:** 10.14814/phy2.13878

**Published:** 2018-09-19

**Authors:** Al Mamun, Utako Yokoyama, Junichi Saito, Satoko Ito, Taro Hiromi, Masanari Umemura, Takayuki Fujita, Shota Yasuda, Tomoyuki Minami, Motohiko Goda, Keiji Uchida, Shinichi Suzuki, Munetaka Masuda, Yoshihiro Ishikawa

**Affiliations:** ^1^ Cardiovascular Research Institute Yokohama City University Yokohama Japan; ^2^ Department of Emergency medicine Graduate School of Medicine Yokohama City University Yokohama Japan; ^3^ Department of Surgery Yokohama City University Yokohama Japan; ^4^ Cardiovascular Center Yokohama City University Medical Center Yokohama Japan

**Keywords:** Abdominal aortic aneurysm, elastic fiber, interleukin‐6, EP4, prostaglandin E

## Abstract

Abdominal aortic aneurysm (AAA) is a progressive disease that has an increasing prevalence with aging, but no effective pharmacological therapy to attenuate AAA progression is currently available. We reported that the prostaglandin E receptor EP4 plays roles in AAA progression. Here, we show the effect of CJ‐42794, a selective EP4 antagonist, on AAA using two mouse models (angiotensin II‐ and CaCl_2_‐induced AAAs) and human aortic smooth muscle cells isolated from AAA tissue. Oral administration of CJ‐42794 (0.2 mg/kg per day) for 4 weeks significantly decreased AAA formation in ApoE^−/−^ mice infused with angiotensin II (1 *μ*g/kg per min), in which elastic fiber degradation and activations of matrix metalloproteinase (MMP)‐2 and MMP‐9 were attenuated. Interleukin‐6 (IL‐6) proteins were highly expressed in the medial layer of angiotensin II‐induced mouse AAA tissues, whereas this expression was significantly decreased in mice treated with CJ‐42794. AAA formation induced by periaortic CaCl_2_ application in wild‐type mice was also reduced by oral administration of CJ‐42794 for 4 weeks. After oral administration of CJ‐42794 beginning 2 weeks after periaortic CaCl_2_ application and continuing for an additional 4 weeks, the aortic diameter and elastic fiber degradation grade were significantly smaller in CJ‐42794‐treated mice than in untreated mice. Additionally, in smooth muscle cells isolated from human AAA tissues, stimulation of CJ‐42794 inhibited PGE_2_‐induced IL‐6 secretion in a dose‐dependent manner and decreased PGE_2_‐induced MMP‐2 activity. These data suggest that inhibition of EP4 has the potential to be a pharmacological strategy for attenuation of AAA progression.

## Introduction

Abdominal aortic aneurysm (AAA) is a relatively common progressive lethal disease among the elderly in most countries (Mani et al. 2011), but there is currently no proven medical therapy. Although recent studies in developed countries indicate that AAA prevalence has declined as a result of changes in lifestyle and pharmacological treatment of cardiovascular risk factors, such as lipid‐lowering drugs (Jacomelli et al. 2016; Stackelberg et al. 2017), the AAA prevalence was still over 1% in men between 65 and 75 years of age (Jacomelli et al. 2016; Stackelberg et al. 2017). A meta‐analysis of AAA demonstrated that AAA prevalence was high in patients with coronary artery disease (8.4%) (Elkalioubie et al. 2015). The number of deaths by aortic aneurysm and dissection *per capita* has been increasing in developing countries (Sampson et al. 2014; Sidloff et al. 2014). These reports suggest that AAA remains a global burden.

Previously, early detection by screening with ultrasound, increased the number of elective open repairs and endovascular aneurysm repairs, which improved the short‐ and long‐term outcome of patients with AAA (Lilja et al. 2017). However, the incidence of intact AAA repair remains consistent (Lilja et al. 2017). Additionally, the 30‐day mortality after ruptured AAA repair remains high (28%) (Lilja et al. 2017). Therefore, a pharmacological strategy to inhibit or slow AAA progression is desired.

Dysregulation of PGE_2_‐mediated inflammatory responses has been suggested to cause AAA. Patient‐derived tissues show high cyclooxygenase‐2 (COX‐2) and PGE_2_ expression (Holmes et al. 1997; Cipollone et al. 2001), and mouse studies showed that COX‐2‐PGE_2_ signaling inhibition or genetic‐depletion attenuated the progression of AAA (Gitlin et al. 2007; Wang et al. 2008). PGE_2_, by binding to its receptors, EP1, EP2, EP3, and EP4, plays pleiotropic roles depending on environmental cues and exerts both pro‐ and anti‐inflammatory effects to maintain homeostasis (Woodward et al. 2011; Yokoyama et al. 2013). It is reported that EP4 expression was upregulated in human AAA tissues and correlated with degeneration of elastic fibers (Yokoyama et al. 2012). A recent study reported that EP4 expression in human AAA was correlated with smoking habit (Dilme et al. 2014), which is a strong risk factor of AAA.

Among PGE_2_ receptors, EP4 is gaining attention as a therapeutic target for some types of chronic inflammation such as rheumatoid arthritis (Sakata et al. 2010). It has been reported that an EP4 antagonist ONO‐AE3‐208 attenuated angiotensin II‐induced AAA in ApoE^−/−^ mice (Cao et al. 2012; Yokoyama et al. 2012), suggesting that inhibition of EP4 signaling has the potential to attenuate AAA progression. Further investigation of the utility of EP4 antagonists in AAA are required.

In the present study, we aimed to examine, using mouse AAA models, whether EP4 antagonist‐mediated AAA attenuation is a class effect, and investigate whether an EP4 antagonist inhibits matrix metalloproteinase (MMP) activation and cytokine release in human smooth muscle cells isolated from patients with AAA (hAASMCs). Here, we demonstrate the effects of the selective EP4 antagonist CJ‐42794 [(S)‐4‐(1‐(5‐chloro‐2‐(4‐fluorophenyoxy) benzamido)ethyl) benzoic acid] on two types of AAA mouse models and hAASMCs.

## Materials and Methods

### Reagents

CJ‐42794 was purchased from Cayman Chemicals (Ann Arbor, MI, USA). Prostaglandin E_2_ (PGE_2_) and indomethacin were purchased from Calbiochem (Billerica, MA) and Tokyo Chemical Industry (Tokyo, Japan), respectively. Anti‐interleukin‐6(IL‐6) antibody and the enzyme‐linked immunosorbent assay (ELISA) for IL‐6 were purchased from R&D System (Minneapolis, USA). Gelatin was purchased from Wako Pure Chemical Industries (Osaka, Japan). Angiotensin II was purchased from Sigma‐Aldrich (St Louis, MI).

### Angiotensin II‐induced AAA

Male ApoE^−/−^ mice (5–6 months of age) were infused with angiotensin II (1 *μ*g/kg per min) for 4 weeks via an osmotic mini pump (Alzet, model 2004, Cupertino, CA, USA), as described previously (Daugherty et al. 2000).

### Calcium chloride‐induced AAA

AAA was induced in wild‐type (C57BL/6) male mice (5–6 months of age) using periaortic application of 0.5 M calcium chloride (CaCl_2_) between the renal arteries and the bifurcation of the iliac arteries, as described previously (Longo et al. 2002).

### Oral CJ‐42794 administration

CJ‐42794 (0.1 mg/kg in 0.5% methyl cellulose) was orally administrated in a volume of 0.5 mL/100 g of body weight twice daily (0.2 mg/kg per day) for 4 weeks. CJ‐42794 was prepared immediately before use. Control group mice received vehicle alone (0.5% of methyl cellulose; FUJIFILM Wako Pure Chemical Corporation, Osaka, Japan).

### Aorta diameter measurement and histological analysis

At the end of the experimental period, mice were euthanized using an overdose of pentobarbital and were perfusion‐fixed with a mixture of 10% buffered‐formalin at physiological perfusion pressure. The abdominal aorta was photographed to determine its external diameter using Image J software. Aortic morphometry was performed by an investigator in a blinded manner, as described previously (Yokoyama et al. 2012).

Formalin‐fixed aortic tissues were embedded in paraffin using a standard protocol. Paraffin embedded cross‐sections of each abdominal aorta (4 *μ*m thick) were stained with elastica van Geison and examined under a light microscope for elastic fiber formation. Elastic fiber degradation was graded as described previously (Sun et al. 2007). Briefly, elastic fiber degradation was graded as follows: grade 1, intact elastin laminae; grade 2, elastic laminae with some interruptions; grade 3, elastic laminae with multiple interruptions and breaks; and grade 4, severe elastin fragmentation or loss.

### Human aortic smooth muscle cells isolated from AAA

We obtained surgical specimens from three individuals with AAA at Yokohama City University and Yokohama City University Medical Center, and isolated human aneurysm aortic smooth muscle cells (hAASMCs), as described previously (Yokoyama et al. 2012).

### Ethics statement

All animal studies were approved by the Institutional Animal Care and Use Committees of Yokohama City University in accordance with the Guide for the Care and Use of Laboratory Animals (reference number: F‐A‐16‐011).

The protocol for using human AAA tissues was approved by the human subject committees at Yokohama City University (reference number: B170800045), and conformed to the principles outlined in the Declaration of Helsinki. Human AAA tissues were obtained from patients during open repair of AAA in Yokohama City University and Yokohama City University Medical Center. All samples were obtained after receiving written informed consent.

### Gelatin zymography

Mouse AAA tissues were freshly isolated at the end of angiotensin II infusion. hAASMCs were plated onto uncoated 96 well plates at 1 × 10^5^ cells/well and serum–starved for 24 h. Cells were then incubated with indomethacin for 1 h to inhibit endogenous PGE_2_ production, followed by stimulation with PGE_2_ (1 *μ*mol/L) with or without CJ‐42794 (1 *μ*mol/L) for 48 h. Gelatin zymography was performed using angiotensin II–infused mouse aortic tissues and culture supernatant from hAASMCs, as described previously (Yokoyama et al. 2012).

### ELISA for IL‐6

hAASMCs were plated onto uncoated 96 well plates at 1 × 10^5^ cells/well and serum–starved for 24 h. Cells were then incubated with indomethacin for 1 h to inhibit endogenous PGE_2_ production, followed by stimulation with PGE_2_ (1 *μ*mol/L) with or without CJ‐42794 for 6 h. The IL‐6 content in mouse AAA tissues isolated at the end of angiotensin II infusion and in culture supernatant from hAASMCs were measured using ELISA (R&D Systems), according to the manufacturer's instructions. IL‐6 protein abundance in aortic tissues was normalized using the total protein concentration determined by the Bradford assay.

### Immunohistochemistry

For IL‐6 immunostaining, an avidin‐biotin‐peroxidase method was used following a standard protocol. Tissue sections were cut from paraffin‐embedded mouse AAA. An anti‐IL‐6 antibody was applied at a dilution of 1:100. Immunohistochemistry and imaging were performed as described (Yokoyama et al. 2006). A color extraction method using BIOREVO bz‐9000 and associated software (KEYENCE, Osaka, Japan) was performed to quantify IL‐6 expression as described (Yokoyama et al. 2014). Entire field of the tunica media of the aorta was examined in each sample. The area stained with diaminobenzidine (DAB) was extracted and counted using the software.

### Statistics

All values are shown as the mean ± standard error of the mean (SEM) of at least three independent experiments. Statistical analysis between two groups was performed using the Mann–Whitney U test. Statistical comparison among multiple groups was performed using the Kruskal–Wallis test, followed by Fisher's least significant difference post hoc test, and the Mann–Whitney U test. A *P*‐value <0.05 was considered statistically significant. All data were analyzed using the GraphPad PRISM software (GraphPad Software, La Jolla, CA).

## Results

### An EP4 antagonist attenuated angiotensin II‐induced AAA

**Figure 1:**
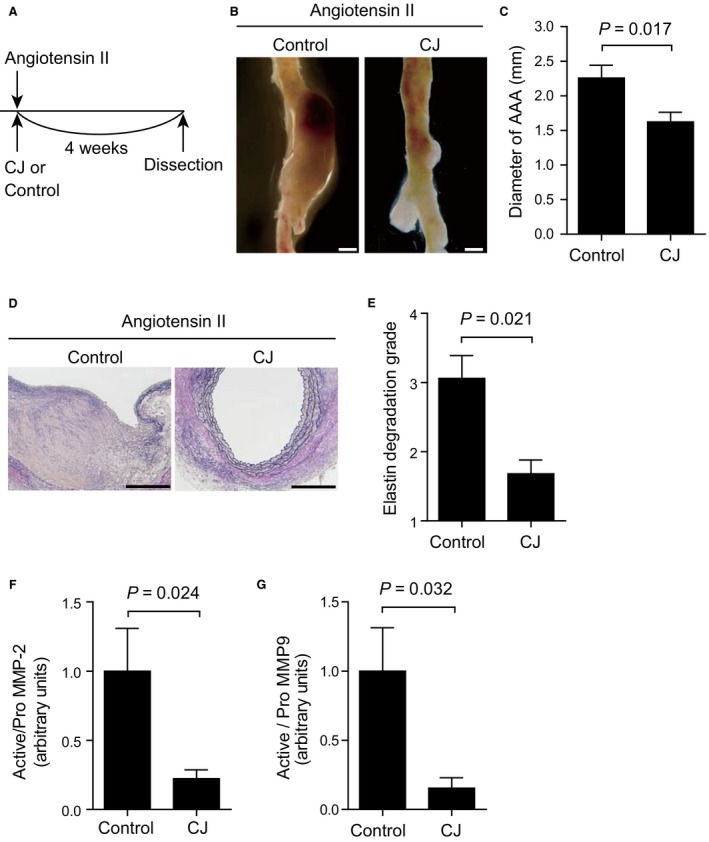
A selective EP4 antagonist CJ‐42794 attenuated angiotensin II‐induced AAA. (A) Time‐course of angiotensin II infusion and CJ‐42794 treatment. (B) Representative images of the abdominal aorta from control and CJ‐42794‐treated (0.2 mg/kg per day) groups (Control and CJ, respectively). Scale bars, 1 mm. (C) Quantification of maximum external diameter of angiotensin II‐induced AAA in ApoE^−/−^ mice. *n *=* *5 (Control) and 6 (CJ). (D) Representative elastica van Gieson stain images of traverse section of angiotensin II‐induced AAA. Scale bars, 200 *μ*m. (E) Medial elastic fiber degradation was graded using an arbitrary scale from grade 1 to grade 4. *n *=* *5 (Control) and 6 (CJ). (F and G) Quantification of MMP‐2 and MMP‐9 activation in mouse AAA tissues measured using gelatin zymography. *n *=* *5 (Control) and 6 (CJ). The data were obtained from two independent experiments.

To examine the effect of CJ‐42794 on aneurysm formation, we use ApoE^−/−^ mice infused with angiotensin II for 4 weeks. CJ‐42794 and angiotensin II administration started at the same time and continued for 4 weeks (Fig. 1A). In the previous report, 0.05 mg/kg per day of ONO‐AE3‐208, an EP4 antagonist, decreased aortic aneurysm formation in angiotensin II‐ and CaCl_2_‐induced mouse AAA (Yokoyama et al. 2012). The inhibitory constant (Ki) value of ONO‐AE3‐208 is 1.3 nmol/L for EP4 (Kabashima et al. 2002) and that of CJ‐42794 is 3.2 nmol/L (Murase et al. 2008). We therefore used 0.2 mg/kg per day of CJ‐42794 as a similar dose to ONO‐AE3‐208. The administration of CJ‐42794 did not affect blood pressure changes induced by angiotensin II (data not shown).

Angiotensin II‐induced prominent AAA formation was observed in a control group, while external aortic diameter was smaller in CJ‐42794‐treated mice compared with control mice (Fig. 1B and C). Elastic fiber formation was more preserved in CJ‐42794‐treated mice, compared with a control group (Fig. 1D and E). Activation of MMP‐2 and MMP‐9, which are elastolytic enzymes, was also significantly decreased in CJ‐42794‐treated mice compared with controls (Fig. 1F and G).

### CJ‐42794 treatment decreased IL‐6 proteins in angiotensin II‐induced AAA

**Figure 2:**
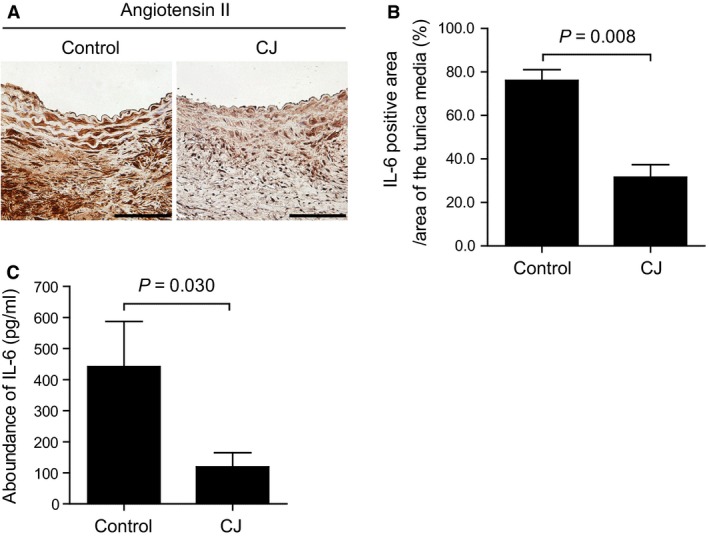
The effect of CJ‐42794 on IL‐6 protein expression in angiotensin II‐induced AAA. (A) Immunohistochemistry for IL‐6 in angiotensin II‐induced AAA treated with or without CJ‐42794 (CJ and Control, respectively). A brown color indicates a positive immunoreaction for IL‐6. Scale bar, 100 *μ*m. (B) Quantification of positive immune‐reactive area for IL‐6 proteins in the tunica media of **A**. *n *=* *5 (Control) and 6 (CJ). (C) Quantification of IL‐6 proteins in mouse AAA using ELISA. *n *=* *5 (Control) and 6 (CJ). The data were obtained from two independent experiments.

Vascular inflammation is a prominent feature of atherosclerotic AAA (Yoshimura et al. 2018). We examined IL‐6 protein expression in angiotensin II‐induced AAA in ApoE^−/−^ mice. Immunohistochemistry showed a strong immune reaction for IL‐6 in the aortic wall in the control group, while this reaction was moderate in CJ‐42794‐treated mice (Fig. 2A). The area of positive immunoreaction for IL‐6 in the tunica media was greater in the control group than in CJ‐42794‐treated group (Fig. 2B). In addition, an ELISA demonstrated that CJ‐42794 administration decreased IL‐6 protein concentrations in the angiotensin II‐treated mouse aorta (Fig. 2C).

### The effect of CJ‐42794 on CaCl_2_‐induced AAA

**Figure 3:**
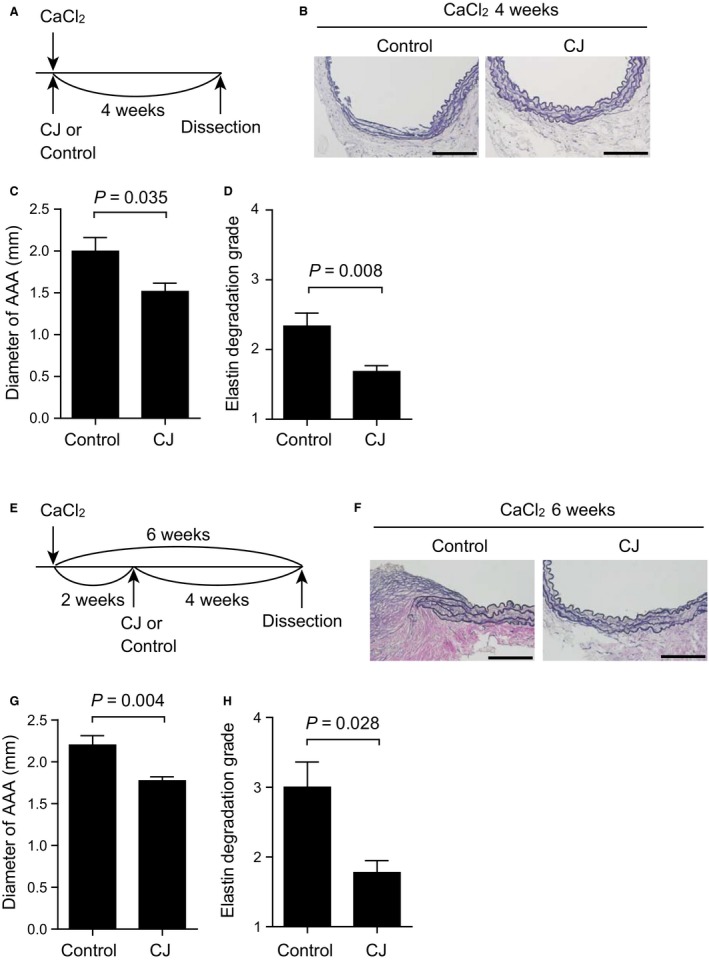
CJ‐42794 decreased CaCl_2_ ‐induced AAA formation. (A) Time‐course of CaCl_2_ application and CJ‐42794 treatment for (B–D) CJ‐42794 administration began at the same day as CaCl_2_ application and continued for 4 weeks (CaCl_2_ 4 weeks). (B) Representative elastica van Gieson stain images of the traverse section of AAA 4 weeks after CaCl_2_ application. Vehicle (control) and CJ‐42794 (CJ)‐treated mice are shown. Scale bars, 100 *μ*m. (C) Quantification of maximum external diameter of AAA 4 weeks after CaCl_2_ application. *n *=* *9 (Control) and 10 (CJ). (D) Medial elastic fiber degradation of mouse AAA 4 weeks after CaCl_2_ application was graded using an arbitrary scale from grade 1 to grade 4. *n *=* *9 (Control) and 10 (CJ). (E) Time‐course of CaCl_2_ application and CJ‐42794 treatment for (F–H). CJ‐42794 administration began 2 weeks after CaCl_2_ application and continued for additional 4 weeks (CaCl_2_ 6 weeks). *n *=* *5–6. (F) Representative elastica van Gieson stain images of traverse section of AAA 6 weeks after CaCl_2_ application. Scale bars, 100 *μ*m. (G) Quantification of the maximum external AAA diameter 6 weeks after CaCl_2_ application. *n *=* *5 (Control) and 6 (CJ). (H) Medial elastic fiber degradation of mouse AAA 6 weeks after CaCl_2_ application was graded the same as in D. *n *=* *5 (Control) and 6 (CJ). The data were obtained from three independent experiments.

We then investigated whether CJ‐42794 administration decreased AAA formation in another mouse model. We examined the effect of CJ‐42794 using CaCl_2_‐induced AAA in wild‐type mice. For the prevention study, CJ‐42794 administration began at the same day as CaCl_2_ application (Fig. 3A). The external diameter of the AAA was decreased in CJ‐42794‐treated mice, compared with a control group (Fig. 3B and C), and elastic fiber degradation was also reduced in CJ‐42794‐treated mice compared with controls (Fig. 3B and D).

CJ‐42794 administration began 2 weeks after CaCl_2_ application, and continued for an additional 4 weeks (Fig. 3E). The AAA external diameter was significantly smaller in CJ‐42794‐treated mice compared with the control group (Fig. 3F and G). The degree of elastic fiber degradation was less in CJ‐42794‐treated mice compared with the saline control group (Fig. 3F and H). These data suggest that inhibition of EP4 using CJ‐42794 attenuates AAA formation in mouse models.

### The effect of CJ‐042794 on aortic smooth muscle cells isolated from human AAAs

**Figure 4:**
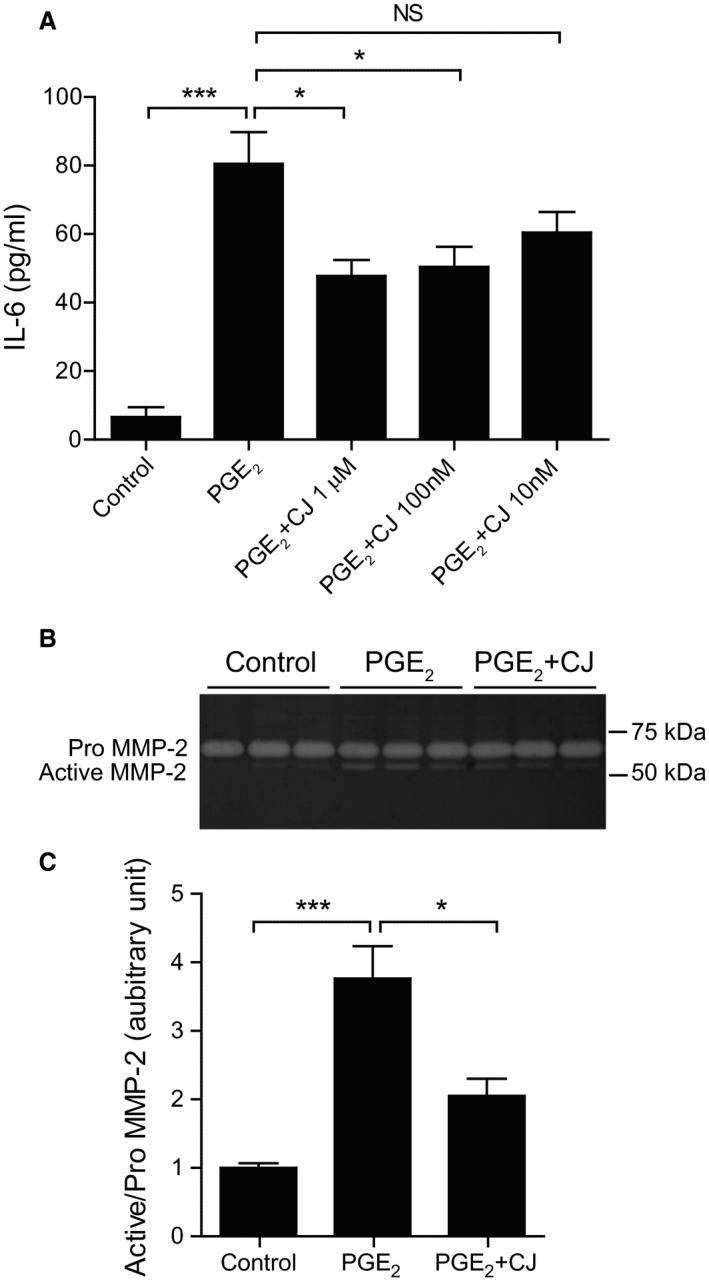
The effect of CJ‐42794 on IL‐6 production and MMP‐2 activation in human aortic smooth muscle cells isolated from AAAs. (A) IL‐6 production in hAASMCs treated with PGE_2_ (1 *μ*M) in the presence or absence of CJ‐42794 for 6 h was measured by ELISA. Data were obtained from three independent experiments using hAASMCs isolated from three individuals with AAA. *n *=* *9, *P <* *0.05; ***P <* *0.001; NS, not significant. (B) Representative images of gelatin zymography. hAASMCs were treated with PGE_2_ (1 *μ*mol/L) or PGE_2_ + CJ‐42794 (1 *μ*mol/L) for 48 h. (C) Quantification of MMP‐2 activation in B. Data were obtained from three independent experiments using hAASMCs isolated from three individuals with AAA. *n *=* *9, *P <* *0.05; ***P <* *0.001; NS, not significant.

Based on the results obtained using mouse AAA models, we examined the effect of CJ‐42794 on hAASMCs. PGE_2_ greatly induced IL‐6 production in hAASMCs, and this effect was attenuated by CJ‐42794 in a dose‐dependent manner (Fig. 4A). Activation of MMP‐2, which is mainly secreted from smooth muscle cells, was increased by PGE_2_ stimulation in hAASMCs, and this elevation was significantly decreased by CJ‐42794 (Fig. 4B and C).

## Discussion

In the present study, we demonstrated that a selective EP4 antagonist CJ‐42794 decreased AAA formation in an angiotensin II‐induced model in ApoE^−/−^ mice, in which elastic fiber degradation and activation of MMP‐2 and MMP‐9, and IL‐6 production were attenuated. CJ‐42794 administration also significantly inhibited AAA formation and elastic fiber degradation in a CaCl_2_‐induced model in wild‐type mice. In human diseased smooth muscle cells, CJ‐42794 stimulation decreased PGE_2_‐induced IL‐6 production and MMP‐2 activation. In previous reports, another selective EP4 antagonist, ONO‐AE3‐208, had an inhibitory effect on angiotensin II‐induced mouse AAA (Cao et al. 2012; Yokoyama et al. 2012). Together with these reports, our data demonstrated that EP4 antagonist‐mediated AAA attenuation is potentially a class effect, instead of a drug–specific effect.

Inhibition of PGE_2_ production by nonsteroidal anti‐inflammatory drug (NSAID) or COX inhibitors attenuated AAA in mouse models (Holmes et al. 1996; Miralles et al. 1999; Armstrong et al. 2005; King et al. 2006). In humans, an observational study demonstrated that the growth of AAA was mitigated in patients taking NSAIDs (Walton et al. 1999). Based on this evidence, PGE_2_ appears to play an important role in AAA progression, and inhibition of the PGE_2_–mediated signaling cascade would be a pharmacological therapeutic strategy. However, clinical studies have shown that COX‐2 inhibition can induce multiple cardiovascular adverse events (Ray et al. 2002; McGettigan and Henry 2006). A major limitation of long‐term NSAID use is damage to the gastrointestinal tract, including gastric mucosal injury (Lanza 1984). Thus, NSAIDs and COX‐2 inhibitors are not suitable for long‐term administration.

Thus, selective inhibition of downstream of PGE_2_ was thought to avoid severe adverse effects. Selective PGE_2_ receptor antagonists have been developed (Zeilhofer and Brune 2006) and CJ‐42794 was developed by Pfizer Inc. as a selective EP4 antagonist (Murase et al. 2008). Some reports suggested that EP4 receptor–mediated signaling pathways are associated with mucosal integrity in the gastrointestinal tract (Yokoyama et al. 2013). Takeuchi et al. (2007) investigated in rats whether CJ‐42794 had a deleterious influence on the gastrointestinal tract compared with indomethacin, a COX‐1 selective inhibitor SC‐560, and COX‐2 selective inhibitor rofecoxib, and found that oral administration of 10 mg/kg per day of CJ‐42794 did not cause any damage to the normal rat gastrointestinal mucosa or to the arthritic rat stomach, and they also did not worsen the gastric ulcerogenic response to stress or aspirin in normal rats. The dose of CJ‐42794 (0.2 mg/kg per day) in our study was lower than that in Takeuchi's paper. Although there is no evidence in humans, this low dose of CJ‐42794 may not have severe adverse effects on the gastrointestinal tract.

AAA is thought to have many different causes, such as hemodynamic stress, aging, and smoking, and subsequent immune and inflammatory responses induce extracellular matrix degradation and vascular smooth muscle cell dysfunction, resulting in AAA progression (Yoshimura et al. 2018). Extensive study using animals with or without genetic modification combined with angiotensin II, elastase, or CaCl_2_ applications revealed potential molecules to target various aspects of the AAA pathological processes (Yoshimura et al. 2018). Current animal models of AAA have the following two disease phases: a model‐specific initial developmental phase and a progression phase, which is similar to human pathology (Lysgaard Poulsen et al. 2016; Senemaud et al. 2017). Although a large number of reports identified potential therapeutic targets to treat patients with AAA, a limited number of them focused on the progression phase in in vivo animal models, and in particular, few used human AAA samples (Yoshimura et al. 2018). In the present study, besides the inhibitory effect of CJ‐42794 on AAA formation, we investigated the effect of CJ‐42794 on the progression phase. Administration of CJ‐42794 starting 2 weeks after CaCl_2_ application significantly decreased the AAA diameter and elastic fiber degradation compared to a control group. Additionally, the use of human AAA smooth muscle cells showed the potential beneficial effects on AAA. These data suggest that inhibition of EP4 signaling attenuates AAA formation.

Randomized clinical trials of pharmacologic interventions for AAA have been conducted (Yoshimura et al. 2018). To date, however, no clinical studies have convincingly confirmed the effect of pharmacological treatment against AAA progression. Hyperlipidemia and renin–angiotensin activation are known to be risk factors for AAA progression (Erbel et al. 2014), but AAA expansion was not associated with hypercholesterolemia in clinical studies (Lindholt et al. 2001; Baxter et al. 2008). The efficacy of renin–angiotensin system‐inhibiting drugs also remains controversial (Malekzadeh et al. 2013; Salata et al. 2018).

The selective EP4 antagonist grapiprant has been approved for the treatment of osteoarthritis pain in dogs (Rausch‐Derra et al. 2016). Recently, a clinical study demonstrated that the selective EP4 antagonist LY3127760 at daily doses of 60 mg to 600 mg was safe and tolerable in healthy subjects during oral dosing for 28 days (Jin et al. 2018). It has been reported that LY3127760 had therapeutic effects on animal models of monoiodoacetate‐induced osteoarthritis, adjuvant arthritis, and migraine headache (Blanco et al. 2016). Based on these developments of EP4 antagonists for clinical use, inhibition of EP4 might be considered to be a potential therapeutic strategy for AAA.

## Conclusions

Despite extensive basic research studies using animals and human AAA specimens and clinical studies to attenuate AAA progression, no pharmacological therapy is currently recognized for clinical use. Patients with small to moderate AAA are simply monitored until surgical intervention. The present study demonstrated, using CJ‐42794, that selective inhibition of EP4 has the potential to attenuate AAA progression.

## Conflict of Interest

None declared.
